# HOSPITAL STAFF AGEISM AND OLDER PATIENT’S SUBJECTIVE VIEWS OF AGING JOINTLY PREDICT FUTURE HEALTH

**DOI:** 10.1093/geroni/igae098.0548

**Published:** 2024-12-31

**Authors:** Yaakov Hoffman, Ehud Bodner, Susanna Cohen, Amit Shrira

**Affiliations:** Bar Ilan University, Ramat Gan, HaMerkaz, Israel; Bar-Ilan University, Ramat Gan, HaMerkaz, Israel; Bar Ilan University, Ramat Gan, HaMerkaz, Israel; Bar-Ilan University, Ramat Gan, HaMerkaz, Israel

## Abstract

Over 66% of those hospitalized in internal wards are adults over 65. While older adults’ subjective views on aging (VoA) have been linked with their own health, less is known in the context of hospitalization. Moreover, to the best of our knowledge, previous studies neither extensively examined effects of departmental ageism on patients’ health indices, nor did they examine interactions between medical staff’s ageist attitudes and patient’s own VoA, in predicting subsequent health. Following, the current study addressed these issues. Hospitalized older adults (N=54, average age 76.24±8.19, range 65-101; 50% female) were assessed in 4 waves. Their experience of hospital ageism during hospitalization (Wave 1) predicted several outcomes two weeks later at Wave 2 (e.g., hospitalization satisfaction, b=-.30, re-hospitalization b=-.24) as well as one’s self-rated health four months later, b=-.36 (Wave 4). This latter link was mediated by the change in attitude towards one’s own aging (at Wave 2, a × b=-4.47, 95% CI [-9.7, -.29]). Findings suggest that reducing medical staff ageist attitudes (e.g., by including courses/workshops on ageism in medical staff training) may both enhance long-term health of older internal ward hospitalized patients and reduce care costs to health systems.

